# Extracellular adherence proteins reduce matrix porosity and enhance *Staphylococcus aureus* biofilm survival during prosthetic joint infection

**DOI:** 10.1128/iai.00086-25

**Published:** 2025-03-21

**Authors:** Mohini Bhattacharya, Tyler D. Scherr, Jessica Lister, Tammy Kielian, Alexander R. Horswill

**Affiliations:** 1 Department of Immunology and Microbiology, University of Colorado School of Medicine 549224, Aurora, Colorado, USA; 2 Department of Pathology, Microbiology, and Immunology, University of Nebraska Medical Center 198515, Omaha, Nebraska, USA; 3 Department of Microbiology, University of Iowa 311821, Iowa City, Iowa, USA; 4 Department of Veterans Affairs, Eastern Colorado Health Care System 19982, Aurora, Colorado, USA; University of California Davis, Davis, California, USA

**Keywords:** MRSA, biofilm, matrix, immune clearance

## Abstract

Biofilms are a cause of chronic, non-healing infections. *Staphylococcus aureus* is a proficient biofilm-forming pathogen commonly isolated from prosthetic joint infections that develop following primary arthroplasty. Extracellular adherence protein (Eap), previously characterized in planktonic or non-biofilm populations as being an adhesin and immune evasion factor, was recently identified in the exoproteome of *S. aureus* biofilms. This work demonstrates that Eap and its two functionally orphaned homologs EapH1 and EapH2 contribute to biofilm structure and prevent macrophage invasion and phagocytosis in these communities. Biofilms unable to express Eap proteins demonstrated increased porosity and reduced biomass. We describe the role of Eap proteins *in vivo* using a mouse model of *S. aureus* prosthetic joint infection. The Results suggest that the protection conferred to biofilms by Eap proteins is a function of biofilm structural stability that interferes with the leukocyte response to biofilm-associated bacteria.

## INTRODUCTION

Methicillin-resistant *Staphylococcus aureus* (MRSA) was the causative agent of approximately 70,000 severe infections and 9,000 deaths in the United States in 2024 (1). Recent studies show that while measures to control hospital-associated bacterial transmission have reduced the occurrence of serious *S. aureus* infections, this success has been slowing (2). Approximately 1–3% of total hip and knee arthroplasties continue to be complicated by infection, resulting in longer hospital stays, higher occurrence of revision surgeries and decreased 5-year survival rates (3, 4). Along with the acquisition of resistance to many currently prescribed antibiotics, the ability of *S. aureus* to form biofilms during chronic infections has made this pathogen a substantial cause of concern with approximately 20% of surgical site infections reported to be associated with *S. aureus* (4, 5). Studies have demonstrated that biofilm-associated bacteria can often tolerate up to 1,000 times the antibiotic concentrations that are found to be effective against planktonic or non-biofilm forms of the same strain (6, 7). Furthermore, biofilms are generally recognized as a distinct lifestyle with uniquely attributable virulence mechanisms (8–11). Bacteria communicate within biofilms via quorum-sensing molecules that allow for the development of shared, public goods (12). A consequence of this is the formation of a protective matrix surrounding the biofilm, consisting of one or more components, including proteins, DNA, and/or polysaccharides (13, 14). Since biofilms are formed under nutritional or environmental stresses, this often allows the pathogen to evade host antimicrobial responses until conditions that are favorable for planktonic growth become available (15). When this occurs, biofilm-associated bacteria disperse from the community and often cause disseminated infections including, but not limited to, serious bloodstream-associated conditions (15–17). Therefore, it is imperative that biofilm phenotypes are considered in the prevention of persistent *S. aureus* infections (18).

One of the major secreted and surface-associated proteins found in the *S. aureus* biofilm matrix is extracellular adherence protein (Eap), which is reported to ubiquitously bind to numerous host proteins as well as bacterial and host DNA (19). Eap is primarily secreted from *S. aureus* but is also described as being able to subsequently bind to the bacterial surface via the activity of a neutral phosphatase and other, yet uncharacterized factors (20). Previous studies with planktonic bacteria attribute important anti-inflammatory and anti-angiogenic properties to Eap during *S. aureus* endovascular infection (21). This protein has been demonstrated to contribute to biofilm formation under conditions of stress, including iron starvation and the presence of serum, but the role that Eap could play as a virulence factor during biofilm growth is currently understudied (22, 23). *S. aureus* also expresses two functional orphans of Eap, EapH1 and EapH2, which were recently reported to protect the bacterium from neutrophil-derived proteases (24, 25).

Macrophages are recognized as essential components of an effective immune response to *S. aureus* in wounds and foreign body-associated infections (26–29). Here, we show that the expression of the three Eap proteins (Eap, EapH1, and EapH2) reduces macrophage invasion and phagocytosis of *S. aureus* biofilm bacteria. These phenotypes are specific to macrophages since neutrophils were relatively unaffected by the presence of Eap. Additionally, using an established murine model of prosthetic joint infection, we show that the inability to express Eap causes a significant reduction in bacterial burdens in the joint as well as surrounding tissue (26, 30). Taken together, these data provide evidence for the role of Eap as a biofilm structural protein that promotes *S. aureus* orthopedic infections.

## RESULTS

### Eap proteins contribute to biofilm biomass and structure

**Fig 1:**
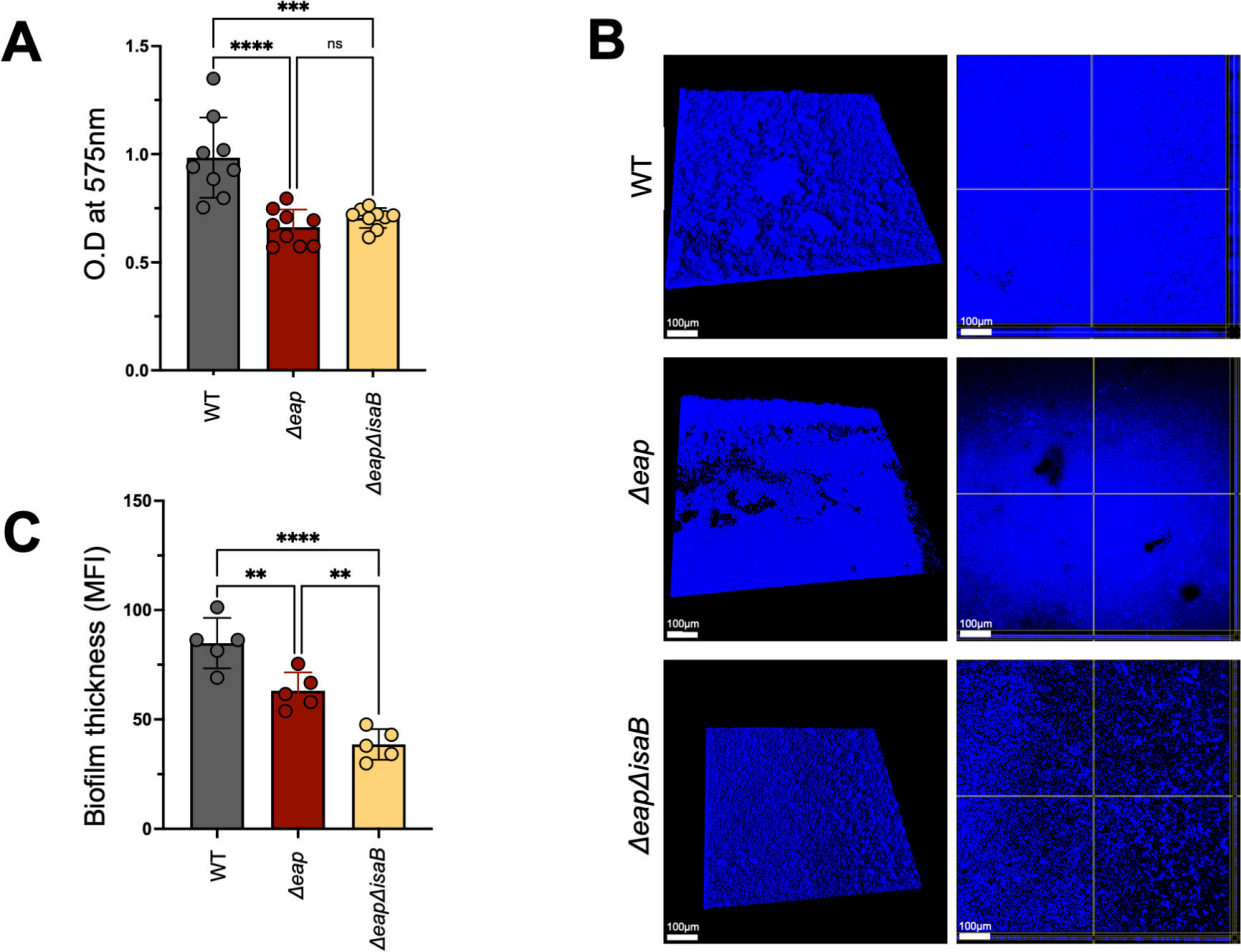
Eap proteins contribute to biofilm biomass and structure. Crystal violet assay measuring biomass of WT, Δ*eap* and Δ*eap*Δ*isaB* biofilms grown overnight in 96-well plates. Crystal violet staining was measured as OD at 575 nm using previously established methods (A). Three-dimensional confocal microscopy images of WT, Δ*eap* and Δ*eapisaB* biofilms grown similarly to (A), in eight-well chamber slides. Bacteria were stained with Hoechst Blue 33342, and images were captured at 400× magnification (left). Images of sections were taken close to the bottom of the biofilm for each strain (right) (B). Volume quantification of biofilms grown as described for (B and C). Data represent three independent experiments performed in triplicate (A) or average of independent images from five separate biofilms, with standard errors of means (SEM) (C). Multiple comparisons were made with one-way analysis of variance and Tukey’s *post hoc* test. ****P < 0.0001; ***P < 0.001; **P < 0.01; ns, not significant. Images were taken using Imaris software. MFI calculations were done using ImageJ software. OD, optical density.

To understand if Eap plays a role in biofilm development, we compared the gross biofilm biomass of the most commonly isolated *S. aureus* lineage, USA300 (hereafter referred to as WT) to an isogenic mutant lacking *eap* as well as its two functionally orphaned homologs, *eapH1* and *eapH2* (hereafter *Δeap*) using an established crystal violet-based assay (31). Biomass comparisons of biofilms from both strains grown for 24 h showed that *Δeap* bacteria have a significant loss of biomass compared to WT biofilms (Fig. 1A). The immunomodulatory protein IsaB is another DNA binding protein that is abundantly expressed as part of the exoproteome in biofilms formed by common clinical strains of *S. aureus* (19, 32). The specific role of isaB during bacterial growth as biofilms remains poorly understood. To evaluate the potential contribution of the DNA-binding activity of isaB to biofilm biomass or structure, we generated a mutant of the *isaB* gene in the Δ*eap* strain background. Bacteria lacking IsaB in addition to the three Eap proteins formed biofilms with biomass comparable to *Δeap* bacteria (Fig. 1A). These results indicate that while the three Eap proteins are important for biofilm structure, the immunomodulatory surface protein IsaB does not significantly contribute to *in vitro* biofilm biomass under these conditions. Confocal microscopy was used to further investigate the differences in gross biomass of 24-h biofilms observed with crystal violet assays. Three-dimensional (3D) images indicated that when compared to WT, *Δeap* and *ΔeapΔisaB* biofilms showed a loss of gross structure and thickness (Fig. 1B). Quantification of biofilm biomass from confocal microscopy confirmed that *Δeap* biofilms have a significant loss of thickness compared to WT biofilms, with further decreases in the isogenic *ΔeapΔisaB* strain (Fig. 1C). Collectively, these data indicate that Eap proteins contribute to the gross biofilm biomass and overall structure of *S. aureus* biofilms, while IsaB may also influence biofilm structural properties but does not seem to affect biomass accumulation.

### Eap proteins reduce the porosity of *S. aureus* biofilms

**Fig 2:**
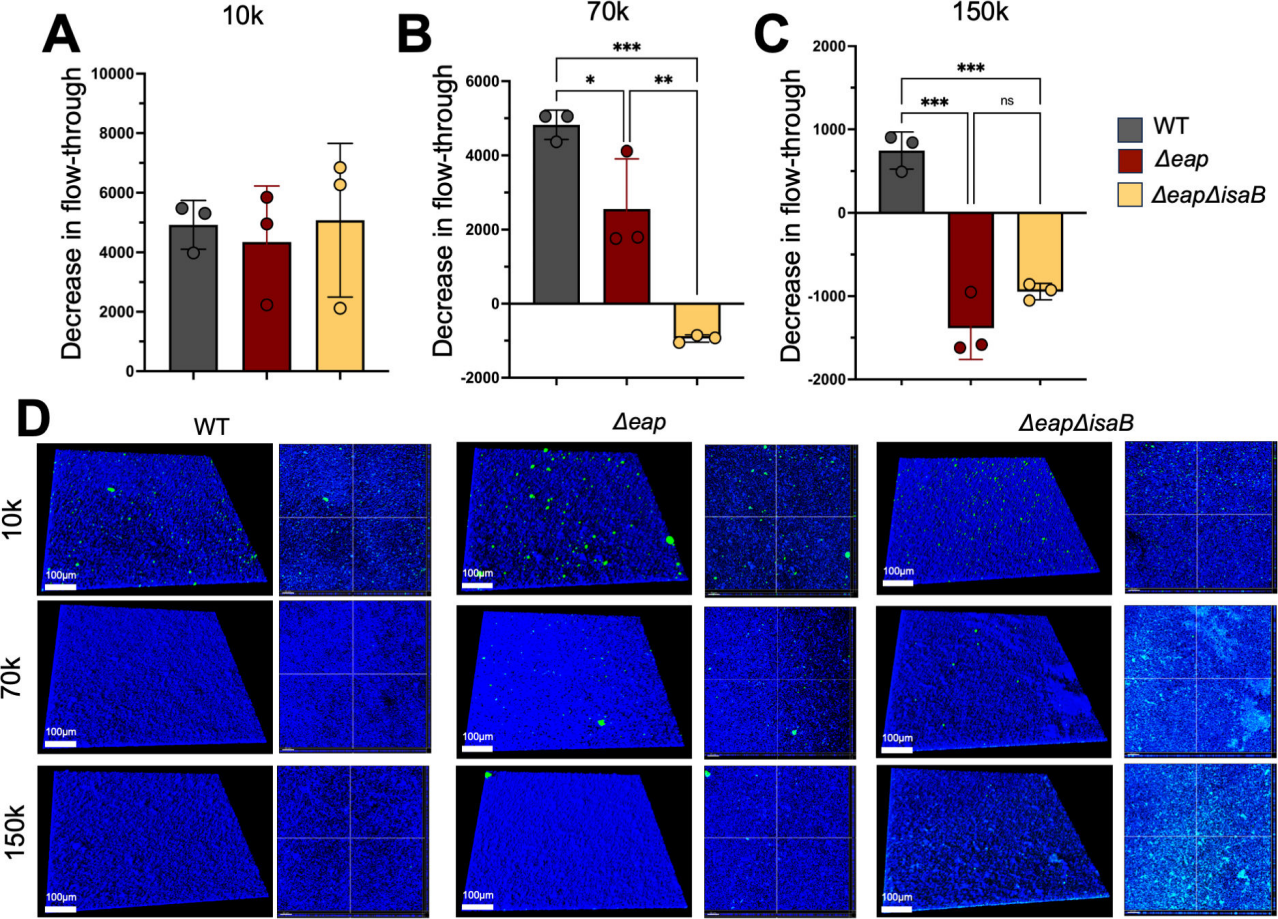
Eap proteins reduce biofilm porosity. Flow through of 10, 70, and 150 kDa FITC-labeled dextran from WT, Δ*eap* and Δ*eapisaB* biofilms grown on 0.45 µm PVDF membranes. Results are calculated in relative fluorescence units as compared to control biofilms lacking dextran (A–C). Representative 3D confocal images of six independent biofilms grown in six-well ibidi chamber slides in duplicate after incubation with 10, 70, and 150 kDa FITC-labeled dextran of respective molecular weights (D). Multiple comparisons were made with one-way analysis of variance and Tukey’s *post hoc* test. ***P < 0.001; **P < 0.01; *P < 0.1; ns, not significant. Images were taken using Imaris software.

To further investigate the role for Eap proteins in providing a specific structural advantage to *S. aureus* biofilms, we tested for differences in porosity when WT biofilms were compared to those formed by the isogenic mutants, *Δeap* and *ΔeapΔisaB*. We utilized three sizes of fluorescein isothiocyanate (FITC)-labeled dextran (10k, 70k, and 150k) and allowed biofilms to grow on 0.45 µm membranes before measuring the levels of each FITC-dextran that could penetrate through biofilms formed by each strain using previously established methods (19). While there were no differences between strains in the levels of 10k FITC-labeled dextran that could penetrate through biofilms (Fig. 2A), we observed a significant increase in the porosity of mutants lacking Eap proteins compared to WT when biofilms were incubated with 70k (Fig. 2B) and 150k FITC-labeled dextran (Fig. 2C). Additionally, we used 24-h biofilms grown in six-channel ibidi flow cells to image the entry and retention of various sizes of FITC-dextran as described above. Confocal images of biofilms incubated with each FITC-dextran for 1 h, followed by three washes in saline revealed that the levels of 70k and 150k (but not 10k) FITC-labeled dextran that could penetrate and be retained in *Δeap* and *ΔeapΔisaB* biofilms were higher than the WT control (Fig. 2D). These data together indicate that Eap proteins prevent the entry of 70–150 kDa particles and therefore contribute to the reduction of the overall porosity of *S. aureus* biofilms, and that the absence of these three proteins increases access to the biofilm.

### Eap proteins contribute to the reduction of macrophage invasion and phagocytosis of *S. aureus* biofilms

**Fig 3:**
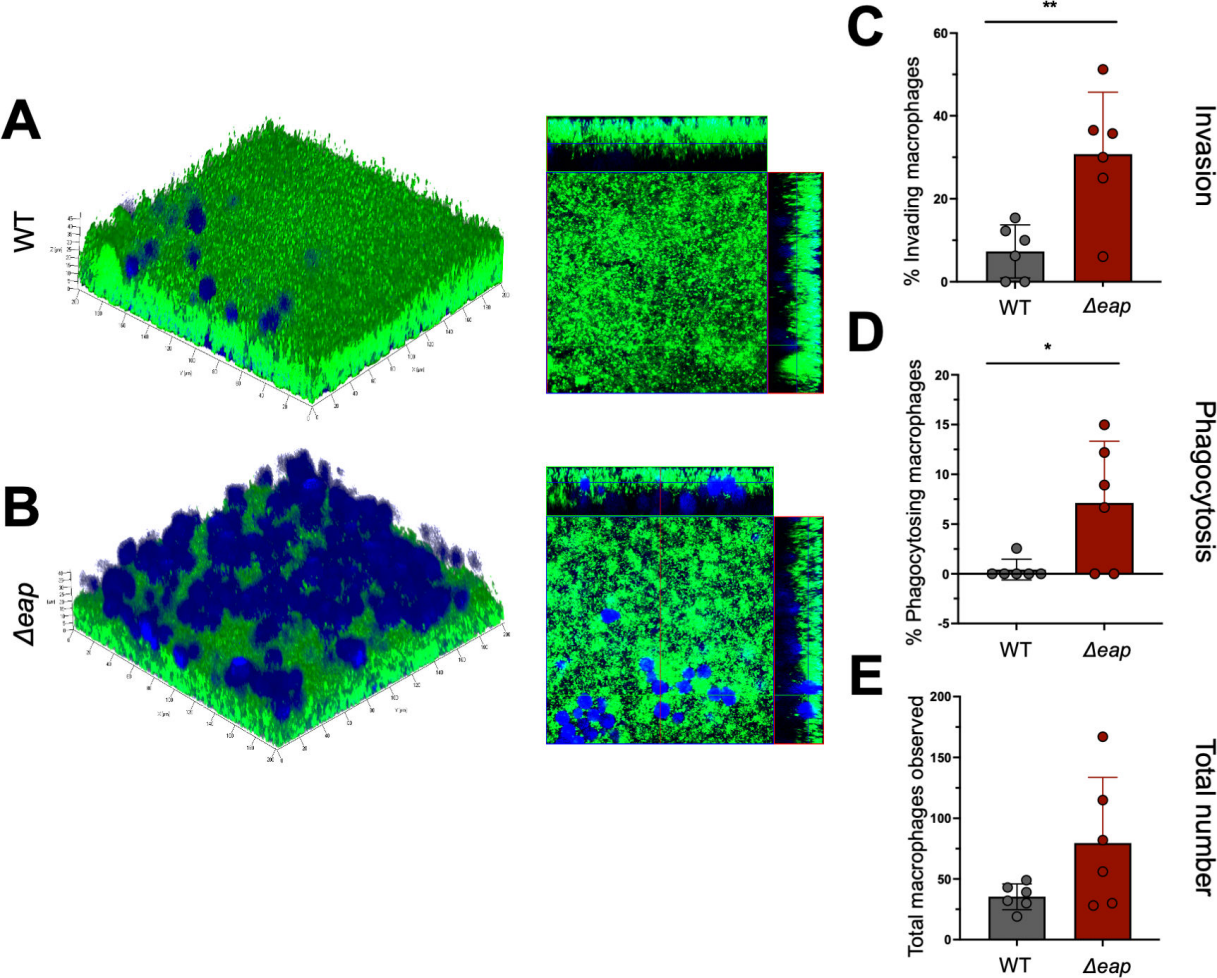
Eap proteins protect biofilms against macrophages. Representative 3D confocal images of green fluorescent protein (GFP)-labeed WT (A) or Δ*eap* (B) biofilms incubated with CellTracker Blue-labeled macrophages for 4–6 h (left). Cross-sectional images from biofilms are shown on the left (right). Quantification of macrophages invading WT or Deap biofilms (C), phagocytosing bacteria (D), and total numbers observed (E) performed from six independent experiments. Student’s *t*-tests were performed for pairwise comparisons (95% CI). **P value = 0.0055; *P value = 0.0261.

Previous reports describe the role of Eap in protecting planktonic *S. aureus* against human neutrophils. While Eap, EapH1, and EapH2 inhibit neutrophil proteases, Eap was shown to bind to neutrophil DNA and interfere with neutrophil extracellular trap (NET) formation (24, 33). Since we found that *S. aureus* biofilms lacking Eap proteins were significantly more porous with reduced biomass. We examined whether these differences would affect the ability of biofilms to evade phagocytosis by innate immune cells (27, 34). Macrophages and neutrophils are required for an appropriate innate immune response to infection (35–37). We therefore incubated mature biofilms with either primary murine bone marrow-derived macrophages or neutrophils for 4–6 h to quantify the number of leukocytes that could penetrate and phagocytose either WT or *Δeap* biofilms. Since we observed a number of significant differences in biofilm properties between the WT and *Δeap* strains, we focused on further evaluating the role of Eap proteins in subsequent assays. Macrophage invasion into *Δeap* biofilms was significantly increased compared to WT, as reflected by both visualization (Fig. 3A and B) and quantification (Fig. 3C). Additionally, numbers of macrophages containing bacteria were also significantly higher in *Δeap* mutant biofilms compared to WT (Fig. 3D) (27). Finally, the total number of observable macrophages associated with *Δeap* mutant biofilms was significantly higher compared to WT, indicating that there were more intact macrophages phagocytosing bacteria from *Δeap* biofilms (Fig. 3E). Collectively, these results demonstrate that Eap proteins are associated with a reduction in invasion and phagocytosis of *S. aureus* biofilms by macrophages.

When similar experiments were performed with primary murine neutrophils, although larger numbers of neutrophils could be observed invading *Δeap* biofilms compared to WT (Fig. S1A and B), this did not reach statistical significance (Fig. S1C). Furthermore, there were no differences in the number of neutrophils observed in phagocytosing biofilm bacteria (Fig. S1D) or the total number of neutrophils present (Fig. S1E), between WT and *Δeap* biofilms. Collectively, these results indicate a larger role for Eap in preventing phagocytosis and clearance of biofilms by macrophages, in comparison to neutrophils.

### Eap proteins contribute to *S. aureus* prosthetic joint infection

**Fig 4:**
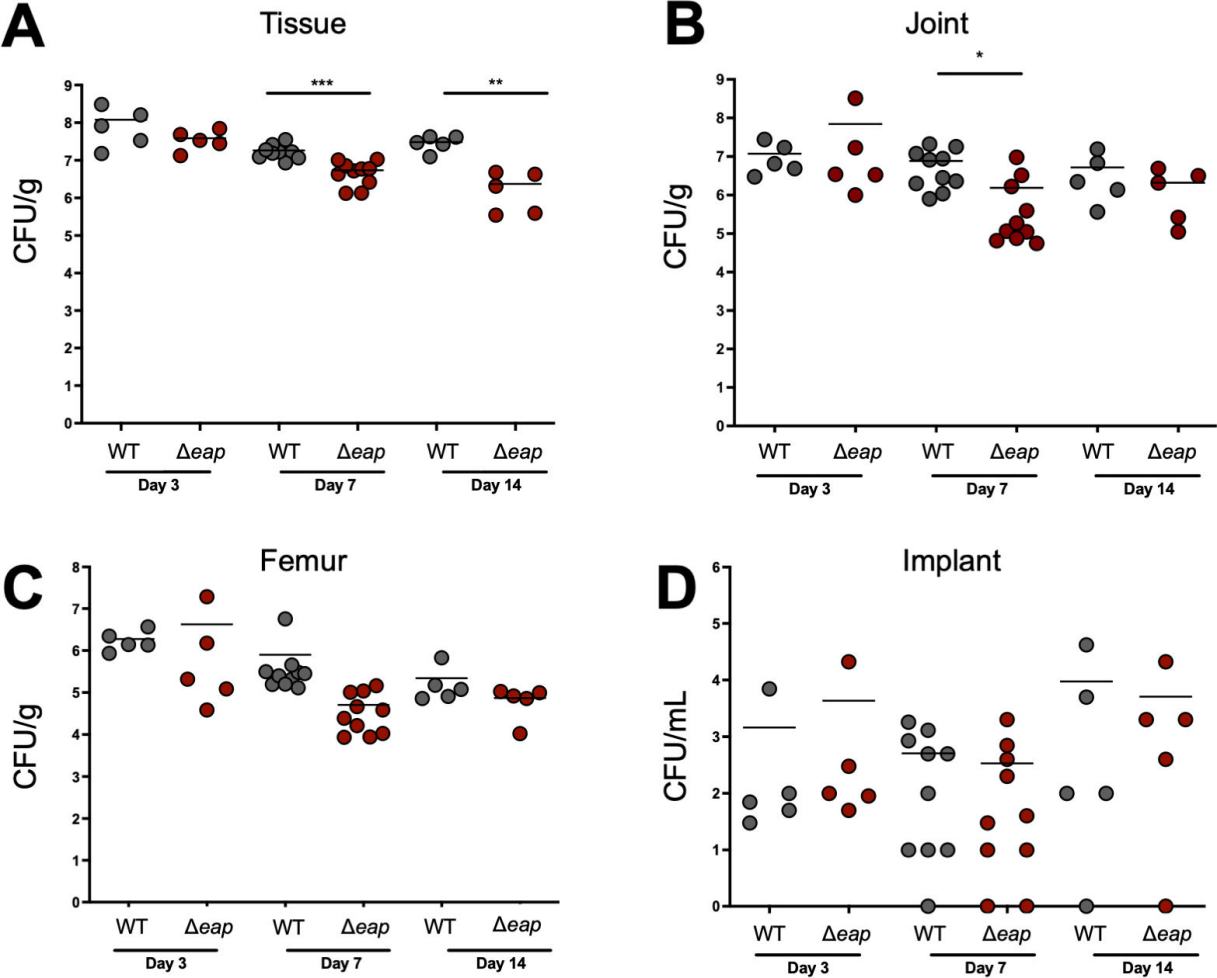
Eap proteins promote bacterial survival during prosthetic joint infection. Bacterial burdens were quantified in C57BL/6 mice infected with WT or Δ*eap S. aureus* at the indicated time points post-infection in the soft tissue surrounding the knee (A), joint (B), femur (C), and sonicated titanium implant (D) (*n* = 5–10 mice/group). Student’s *t*-test was performed for pairwise comparison (95% CI). ***P value = 0.0001, **P value = 0.0061, *P value = 0.0239.

Since biofilms lacking Eap proteins were more susceptible to invasion and phagocytosis by macrophages *in vitro* and exhibited less structural organization*,* we next examined whether these phenotypes would translate to altered biofilm survival *in vivo*. A previously established mouse model of prosthetic joint infection was used to compare the ability of WT and *Δeap* bacteria to form biofilms (38, 39). Three time points were selected to reflect planktonic growth (day 3), transition to biofilm formation (day 7), and chronicity (day 14) based on recalcitrance to systemic antibiotics (29). A larger number of animals was analyzed at day 7 since this represents the transition period to biofilm growth and was considered the best interval to interrogate potential phenotypes given the biofilm structural defects observed with *Δeap in vitro. Δeap* bacterial burden was significantly reduced in the tissue surrounding the infected joint at days 7 and 14 post-infection, which extended to the joint at day 7 with no further reductions at day 14 (Fig. 4A and B). Titers in the femur were also lower at days 7 and 14 with *Δeap*, although this did not reach statistical significance, and no differences were observed on the implant (Fig. 4C and D). Previous work has described the role of granulocytic myeloid-derived suppressor cells (G-MDSCs) in promoting *S. aureus* biofilm survival by their ability to inhibit macrophage proinflammatory activity, neutrophil antimicrobial activity, and T cell activation (26, 38, 40). Therefore, flow cytometry was performed on infected tissue samples to quantify G-MDSC infiltrates in WT- and *Δeap*-infected mice (41). Although the overall number of CD45^+^ leukocytes trended higher in WT-infected mice compared to those infected with *Δeap* bacteria (Fig. S2A), G-MDSC infiltrates (CD45^+^Ly6G^+^Ly6C^+^) were similar between the groups (Fig. S2B). Since neutrophils are also recruited to infected tissues, we measured the number of neutrophils (CD45^+^Ly6G^+^Ly6C^−^) in these animals (Fig. S2C). While *Δeap*-infected mice had lower neutrophil numbers compared to WT at day 7, these differences were not statistically significant at day 14. Altogether, these data suggest that Eap proteins play specific roles in promoting *S. aureus* survival during biofilm-associated infection *in vivo*. While G-MDSC and neutrophil responses were generally comparable between WT- and *Δeap*-nfected conditions, the consequence of this response to bacterial survival is likely altered as a function of Eap expression and involves more complex changes in the macrophage response to infection, as based on our *in vitro* findings.

## DISCUSSION

**Fig 5:**
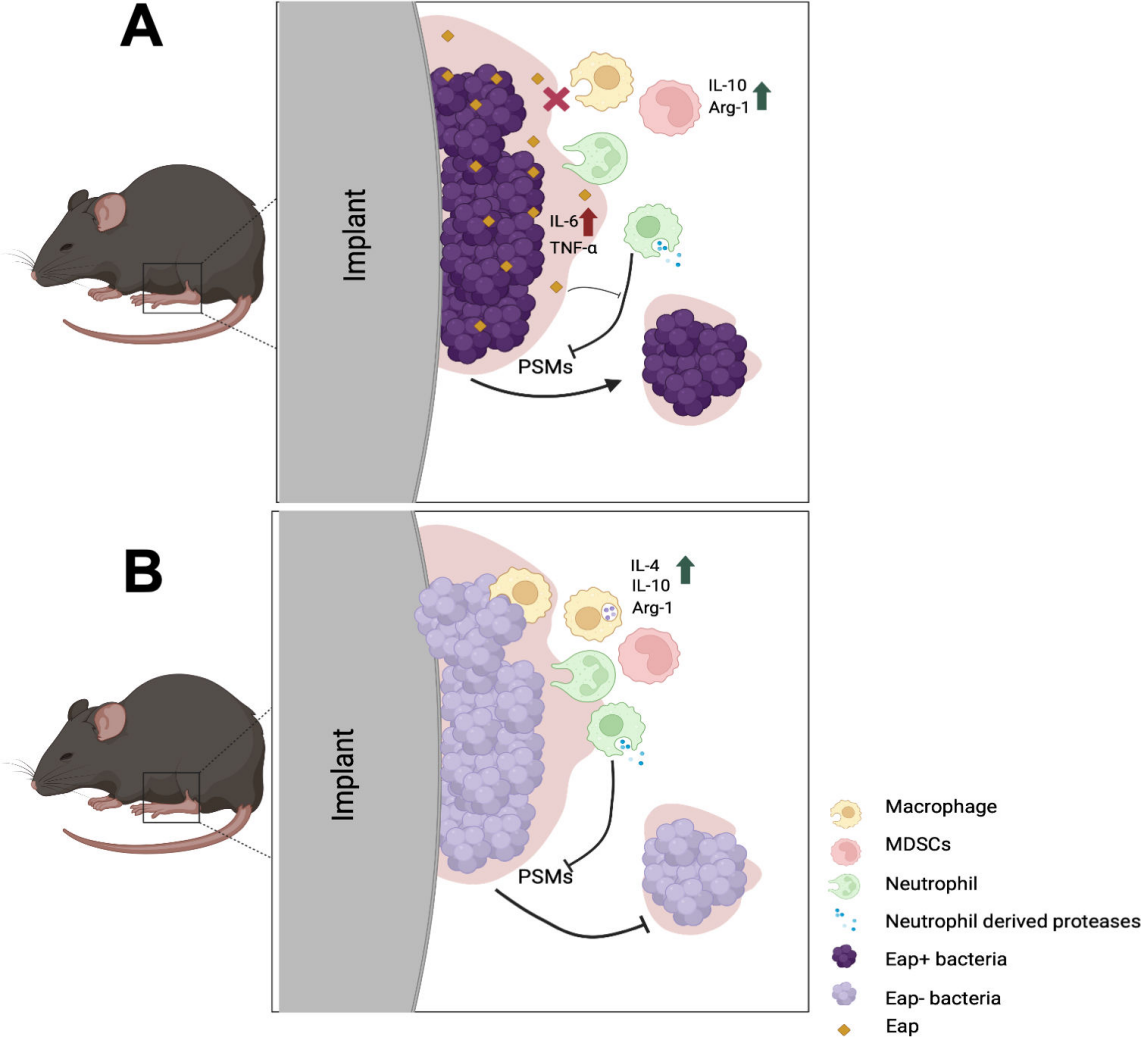
Summary and hypotheses based on current findings and previous literature. Expression of Eap protects biofilms from macrophage invasion and phagocytosis and, to a lesser extent, neutrophils. Proinflammatory signatures associated with Eap expression are likely dampened by the anti-inflammatory macrophage response to biofilms. Eap proteins prevent protease-mediated degradation of phenol-soluble modulins (PSMs), which allows for subpopulations of the community to disperse and spread (A). In the absence of Eap, phagocytes gain some entry into the biofilm and likely phagocytose and kill bacteria. Proteases released from neutrophils degrade PSMs and prevent dispersion, allowing the immune response to continue clearance of the biofilm infection. Inflammation is reduced in the presence of anti-inflammatory signatures generated from biofilm-exposed macrophages (B).

This work provides evidence that Eap proteins are important to *S. aureus* biofilm structure and can influence the host response to infection. We demonstrate that Eap proteins increase the thickness (Fig. 1) and reduce the porosity (Fig. 2) of *S. aureus* biofilms. Absence of these proteins affects macrophage antimicrobial functions (Fig. 3) but does not provide any major advantage when biofilms are exposed to neutrophils. (Fig. S1). *In vivo*, while Eap proteins do not seem to alter the innate immune response to *S. aureus* biofilm infection, bacterial survival was significantly reduced with *Δeap* compared to WT bacteria (Fig. 4). When taken together with our *in vitro* findings, this suggests that Eap proteins may serve to prevent bacterial clearance by phagocytes *in vivo* (Fig. 5).

Recent studies have characterized the serine protease inhibition activity of all three Eap proteins against the neutrophil-derived antimicrobials, neutrophil elastase and Cathepsin G, both of which have demonstrated roles as anti-staphylococcal proteins (34, 42–44). Eap proteins were found to be important for the survival of *S. aureus* injected into the bloodstream (24). It is therefore plausible that this protease inhibition activity prevents neutrophils from clearing *S. aureus* at the earlier stages of infection and depends on the presence of extracellular matrix binding partners such as fibrinogen and collagen, as well as the expression of neutrophil targeting toxins, allowing the subsequent development of biofilms *in vivo*. While our *in vivo* model is an established method to study *S. aureus* biofilm characteristics, the biofilm proteome and subsequent immune response to infection will vary based on tissue niche (11). Additional biofilm-associated infection models might therefore show a larger involvement of neutrophils and must be taken into consideration for future studies. Time course experiments to follow the progression of bacterial survival and biofilm development in the presence of primary neutrophils will also provide insight into this process. Additionally, the role of this anti-protease activity against macrophage antimicrobial proteins also remains to be understood.

Previous reports have shown that Eap proteins are anti-inflammatory and immunomodulatory, with anti-protease activity specifically against human neutrophils. Eap is also known to prevent the degradation of phenol-soluble modulins (PSMs) by neutrophil-derived proteases (45). PSMs can lyse neutrophils and are released during the transition of biofilms to planktonic growth, making them an important virulence factor during infection (46). PSMs are also reported to form amyloid fibers that can stabilize biofilm structure (47). Whether PSMs contribute to Eap-associated tolerance of neutrophils warrants further investigation. Similarly, while studies report a role for Eap in binding to DNA and blocking NET formation, *S. aureus* biofilms are documented to induce NETosis in a leukocidin-dependent manner and to utilize a nuclease to degrade the DNA released from neutrophils (33, 34, 48). Since experiments with Eap were performed with purified protein and chemically induced NETs, analyzing the effect of Eap proteins on NETs released from biofilms would provide more information on the role of Eap proteins during neutrophil ET release (33). Additionally, it has been demonstrated that once Eap binds DNA, it does not cleave it (33). It is therefore possible that Eap-bound host DNA can act as an immune evasion strategy *in vivo*, allowing *S. aureus* to appear as a “self” molecule to the immune system, although this remains speculative (24, 34, 42–44).

Here, we demonstrate that Eap proteins can provide *S. aureus* with some degree of protection against macrophages *in vitro*. It is unlikely that these phenotypes are due solely to the increased access afforded to phagocytic cells by virtue of a reduction in biofilm thickness and porosity. Whether Eap itself has a direct effect on macrophages therefore warrants further investigation. We speculate that although macrophages were found to more effectively invade biofilms *in vitro,* this likely would not have translated to altered recruitment *in vivo*. This is based on our prior observations where the activation status of infiltrating leukocytes in this model is typically a better predictor of whether effects on biofilm burden are observed (49, 50). Therefore, it remains possible that macrophages, and even granulocytes, are polarized to a more proinflammatory state in the absence of *S. aureus* Eap, although this remains speculative. Eap expressed by planktonic bacteria interacts with peripheral blood mononuclear cells presumably via the intercellular adhesion molecule 1 to induce proinflammatory cytokine production (IL-6 and TNF-α) (51). Biofilms bias macrophages toward an anti-inflammatory phenotype (arginase-1, IL-4, and IL-10) that is compounded by the action of immune suppressive G-MDSCs known to impair T cell activation (27, 52). These biofilm-specific mechanisms of macrophage subversion may therefore neutralize any pro-inflammatory signals generated as a function of Eap. Conversely, a number of reports provide evidence that Eap impairs neutrophil and T cell recruitment as well as T cell activation. These functions were attributed to higher concentrations of Eap such as those that would be produced by bacterial biofilms (21, 53, 54). It is therefore likely that the anti-inflammatory properties of Eap are more relevant during biofilm infections, whereas pro-inflammatory processes are associated with the survival of planktonic populations. Similarly, neutrophils may play a more significant role at the early stages of biofilm-associated infections, with Eap influencing the recruitment and cytokine signaling of neutrophils in the prosthetic joint environment. Further studies are required to assess the timeline of primary *in vivo* biofilm establishment in greater detail.

Eap has been found to be important for biofilm formation under conditions of iron starvation, such as would be observed *in vivo*, a process that is regulated by Fur and shown to require the Agr, SaeRS, and SarA regulatory networks, all of which can have significant effects on biofilm formation (22). Additionally, the polysaccharide intercellular adhesin, which forms the primary polysaccharide content in *S. aureus* biofilms, has also been demonstrated to be important for the expression of Eap. It is therefore plausible that the difference observed in the structural integrity of Δ*eap* biofilms is a consequence of the absence of relevant interactions between Eap and additional matrix components. The presence of human serum enables the activity of a neutral phosphatase which assists the redocking of Eap onto the bacterial surface and inter-protein interactions, which contributes to bacterial aggregation and may therefore play a role in the early stages of biofilm development (55). Finally, in addition to DNA, Eap proteins have been documented to promiscuously bind multiple host-associated ligands including fibrinogen and collagen. Synovial fluid is an ultrafiltrate of blood plasma that encases joints and periprosthetic implants (56–58). Whether Eap promiscuously binds to components of synovial fluid is currently unknown. This viscous fluid is known to harbor *S. aureus* aggregate biofilms reported to bind fibrinogen via its two sortase-anchored fibronectin-binding proteins FnbpA and B (59). The properties of these biofilms are distinct from their surface-associated counterparts and can be formed by subpopulations of detached biofilm bacteria (60). It is therefore plausible that the Eap proteins, FnbpA and FnbpB could contribute to biofilm survival at different phases of the infection lifecycle and require the additional activities of dispersion cues, including PSMs, to evade the immune response during prosthetic joint infection.

Due to its prevalence among strains of *S. aureus,* the use of Eap as a marker of infection has been proposed (61). Our study demonstrates that Eap is a potential virulence factor preventing macrophage functions and likely plays additional roles during biofilm infections *in vivo*. A deeper understanding of the tissue-niche specificity of Eap-associated virulence properties, with emphasis on the comprehensive immune response generated to Eap-expressing bacteria as well as the Eap protein itself, has the potential for the development of a monoclonal antibody to target biofilm infections *in vivo*. Additionally, Eap has been characterized as a neutrophil serine protease inhibitor (44). While our results do not show a significant role for Eap in the phagocytosis of biofilms by neutrophils, understanding the interplay between Eap, neutrophil antimicrobial proteins as well as the interaction with additional matrix-associated factors (PSMs, polysaccharide, leukocidins, etc.) is necessary in order to gain insights into the potential use of Eap as an intervention for inflammation that is often associated with *S. aureus* biofilm infections. The concerted effect of Eap and additional biofilm-associated factors may also result in larger impacts on bacterial burdens observed in our *in vivo* model (27, 62, 63).

Altogether, this work builds on previous studies and adds to our knowledge of the innate immune response to *S. aureus* biofilm infections. Figure 5 summarizes our findings and hypotheses based on current and previous work to depict how Eap proteins may be playing a multifactorial role during *S. aureus* biofilm-associated prosthetic joint infection, with potential new avenues of investigation to better understand the complex dynamics that make *S. aureus* a successful biofilm pathogen.

## MATERIALS AND METHODS

### Bacterial strains and growth conditions

Unless otherwise indicated, all experiments were performed in the USA300 clinical strain background. Bacterial cultures were grown in tryptic soy broth (TSB) at 37°C with shaking (200 RPM).

### Construction of *S. aureus* bacterial mutants

Chromosomal deletions of the three Eap encoding genes (*eap*, *eapH1*, and *eapH2*) were performed using previously established methods (19). Briefly, the temperature-sensitive pJB38 plasmid was used to introduce DNA fragments (~1 kb) flanking the target region of interest. Flanking DNA was amplified (Phusion high-fidelity polymerase, NE Biolabs) using gene-specific primers; products were digested with restriction enzymes and purified (Qiagen PCR purification). Following triple ligation into pJB38, the plasmid was electroporated into *Escherichia coli* DC10b and selected Luria Bertani agar plates containing 100 µg/mL ampicillin. Following confirmation from single colonies, plasmid was purified, PCR was used for confirmation with construction and sequencing primers performed, and plasmid was electroporated into *S. aureus*. Positive clones were selected on tryptic soy agar (TSA) containing 10 µg/mL chloramphenicol and homologous recombination performed at 42°C for 24 h. Following overnight incubation in TSA-Cam and a series of subcultures in TSB at 30°C, counterselection was performed on 200 ng/mL anhydrotetracycline at 30°C overnight. Loss of plasmid was indicated by growth on TSA but not TSA-Cam, and the presence of desired mutations was verified using PCR with chromosomal primers that were outside the region of mutation.

### *In vitro* 24-h biofilm growth

All *in vitro* biofilms used for biomass and matrix porosity measurements were grown in TSB containing 0.4% glucose as previously published, unless otherwise indicated (19, 64). Bacterial cultures were grown overnight (16–18 h) in TSB at 37°C with shaking (200 RPM). The next day, bacteria were subcultured (1:100) in fresh TSB for 2–3 h and brought to exponential phase corresponding to an optical density (OD) at 600 nm of 0.5–0.7 as previously described. Cultures were then centrifuged at 3,900 RPM for 2 min, washed once with phosphate-buffered saline (PBS), centrifuged, and resuspended in TSB containing 0.4% glucose for biofilm growth measurements.

### Biofilm biomass measurements using crystal violet staining

Cultures were prepared as described above. Bacteria were seeded into 96-well microtiter plates (Costar, 200 µL per well) and incubated overnight at 37°C in a humidified chamber for 24 h. Biofilms were washed with double distilled water (dd water) and incubated with 0.1% crystal violet for 30 min at room temperature. Crystal violet was drained, and the plate was washed in dd water three times followed by the addition of 33% acetic acid to the wells. After a 30-min incubation, solubilized biofilms were pipetted into a new 96-well plate, and OD was measured at 575 nm. Measurements were made in comparison to the well containing PBS.

### *In vitro* biofilms for confocal imaging

Cultures were prepared in TSB containing 0.4% glucose as described above. Bacteria were seeded into eight-well ibidi μ-slides (ibidi, Cat. No. 80826) and incubated for 24 h at 37°C in a humidified chamber. Spent media were removed, biofilms were washed with PBS and stained with 10 µg/mL Hoechst Blue 33342 stain (Thermo Fisher, Cat. No. H3570) for 30 min for confocal imaging. Biofilms were then washed again with PBS and fixed with 10% formalin. Biofilms were visualized using the Olympus FV1000 confocal laser scanning microscope using the Z-stack feature to collect 3D images spanning the thickness of the biofilm. All experiments were performed with two technical duplicate biofilms per strain for a total *n* = 4 (*n* = 8 biofilm technical replicates per strain). Three images were taken per technical biofilm replicate (*n* = 24 images per strain).

### Measuring porosity of *in vitro* biofilms

Twenty-four-hour biofilms of WT or respective isogenic mutants were grown as described above in 96-well plates containing 0.45 µm PVDF membranes as previously described (19). Briefly, biofilms grown in 96-well plates without a membrane were used as a negative control. Following 24-h growth, control biofilm biomass was measured using the crystal violet assay described above. Media were removed from filter plates and replaced with 100 µL MES (2-(N-morpholino)ethane sulfonic acid) (MES) buffer containing 1 mg/mL FITC-isocyanide-dextran (with dextran at a molecular weight of either 4k, 10k, 70k, or 150k). These experiments were performed using a negative control consisting of biofilms resuspended in buffer lacking FITC-dextran. Filter plates were centrifuged for 45 s at 20 g, flow through collected, and relative levels of fluorescence measured with excitation and emission wavelengths of 470 and 523 nm, respectively. Values were plotted in comparison to the media-only control as a measure of maximum fluorescence. For microscopy, bacteria were grown as described above and seeded into eight-well ibidi μ-slides (ibidi, Cat. No. 80826). Biofilms were washed in PBS and resuspended in 1 mg/mL FITC-isocyanide-dextran of various sizes as described above, for 1 h. 3D images were taken using the Olympus FV1000 system.

### *S. aureus* biofilm-leukocyte co-culture experiments

Confocal microscopy experiments depicting the interaction of macrophages or neutrophils with *S. aureus* biofilms were performed as previously published (27). Briefly, green fluorescent protein (GFP)-labeled bacteria were grown to the exponential phase as described above and seeded into chamber slides coated with human plasma. Biofilms were allowed to grow for 4 days at 37°C and incubated with CellTracker Blue-labeled bone marrow-derived macrophages or thioglycolate-elicited neutrophils from C57BL/6 mice for 4–6 h using a Zeiss laser scanning confocal microscope (LSM 710 META; Carl Zeiss). 3D images of biofilms were collected using Xen 2007 software (Carl Zeiss) as previously described (27). The number of leukocytes invading and phagocytosing biofilms was quantified by measuring the distance of immune cells from the biofilm base (invasion) and by counting the leukocytes containing intracellular bacteria in each field of view using orthogonal images.

### Mouse prosthetic joint infection model

*S. aureus* biofilm infection was studied *in vivo* using an established model of implant-associated prosthetic joint infection. Briefly, 8- to 10-week-old male and female C57BL/6 mice (*n* = 5–10 mice/time point/strain) were used to introduce an implant into the intramedullary canal of the femur as previously described (26, 30, 38, 41). Approximately 10^3^ of WT or *Δeap* bacteria were inoculated at the implant tip, and animals were administered buprenorphine slow release after surgery for pain relief. Animals were euthanized at days 3, 7, and 14 post-infection to collect tissue and implant samples as previously described (41). Tissue homogenates and sonicated implants were plated on TSA containing 5% sheep blood to quantify total colony-forming units (CFU) per gram of tissue or per mL diluent for implants. The soft tissue surrounding the knee was collected to quantify G-MDSC and PMN infiltrates by flow cytometry using antibodies for CD45, Ly6G, and Ly6C as previously described (41).

## Data Availability

Further inquiries and information on reagents and material reported should be directed to the corresponding author. Data can be made available upon request.

## SUPPLEMENTAL MATERIAL

Supplemental material iai.00086-25-s0001.docx Fig. S1 and S2.

ASM does not own the copyrights to Supplemental Material that may be linked to, or accessed through, an article. The authors have granted ASM a non-exclusive, world-wide license to publish the Supplemental Material files. Please contact the corresponding author directly for reuse.
