# Supplementary material for: Extracellular adherence proteins reduce matrix porosity and enhance Staphylococcus aureus biofilm survival during prosthetic joint infection
Source: Infect Immun. 2025 Mar 21;93(4):e00086-25. doi: 10.1128/iai.00086-25 (PMC11977312; doi:10.1128/iai.00086-25)
Supplement: Supplemental material — Fig. S1 and S2. [file iai.00086-25-s0001.docx]

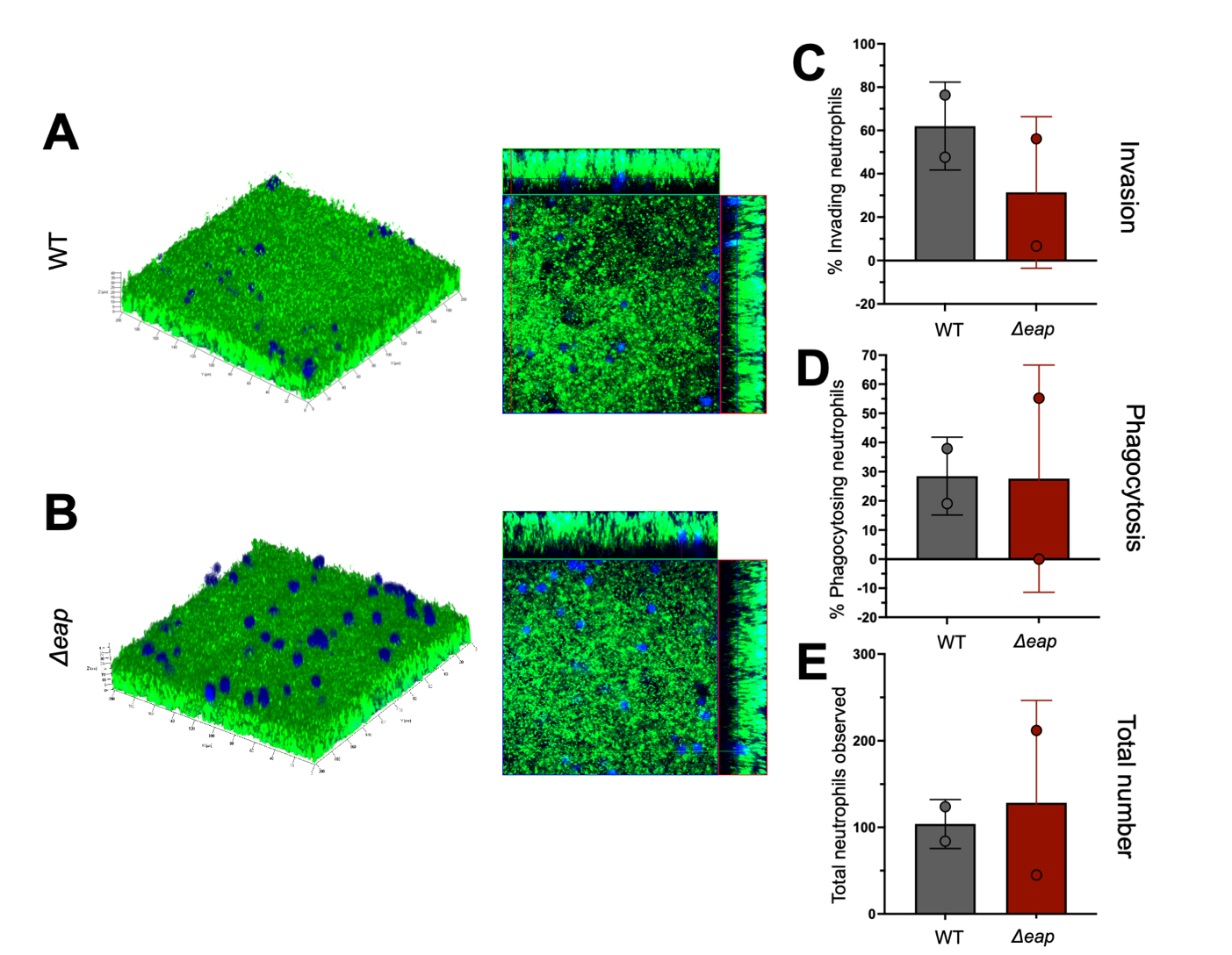
 **Figure S1. Eap proteins do not influence neutrophil responses to biofilm.** Representative 3D confocal images of green fluorescent protein (GFP)-labelled WT (A) or *eap* (B) biofilms incubated with Cell Tracker Blue labelled neutrophils for 4-6 hours (left). Cross sectional images from biofilms shown on left (right). Quantification of neutrophils invading WT or *eap* biofilms (C), phagocytosing bacteria (D) and total numbers observed (E). Student’s test was performed for pair-wise comparison.


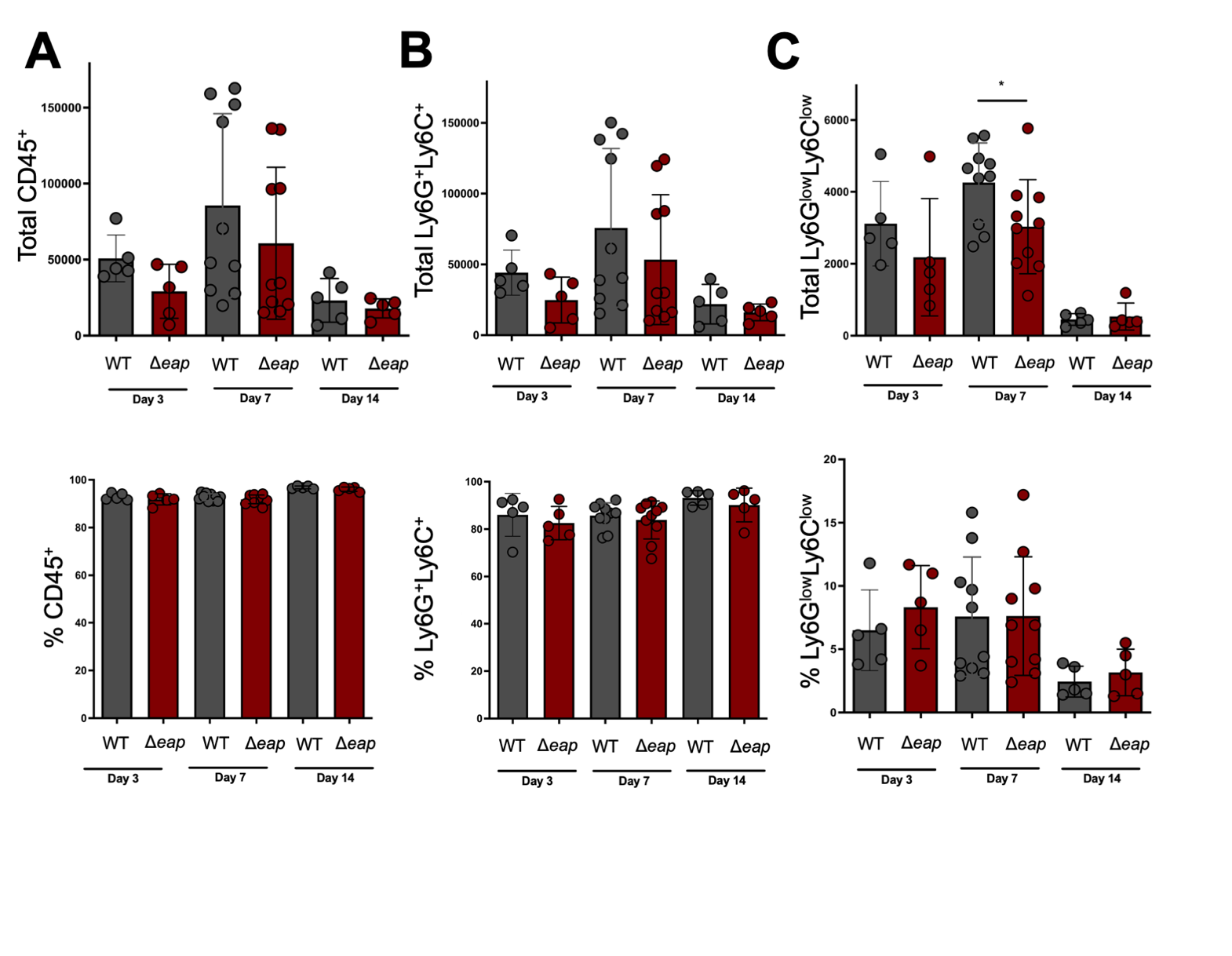


**Figure S2.** **Eap proteins do not affect granulocyte recruitment *in vivo.*** Flow cytometry quantification of CD45^+^ populations (A), granulocytic myeloid-derived suppressor cell (defined as CD45^+^Ly6G^+^Ly6C^+^) (B) and neutrophil (defined as CD45+ Ly6C^low^ Ly6G^low^) (C) populations in tissue homogenates of animals infected with either WT or *eap* bacteria at days 3, 7 and 14 post-infection. Data is presented as total numbers and percentage of each total population. Student’s test was performed for pair-wise comparison. *P value= 0.0366
